# Unique Regulation of Na-K-ATPase during Growth and Maturation of Intestinal Epithelial Cells

**DOI:** 10.3390/cells8060593

**Published:** 2019-06-15

**Authors:** Niraj Nepal, Subha Arthur, Uma Sundaram

**Affiliations:** Department of Clinical and Translational Sciences and Appalachian Clinical and Translational Science Institute, Joan C. Edwards School of Medicine, Marshall University, 1600 Medical Center Drive, Huntington, WV 25701, USA; nepal@marshall.live.edu (N.N.); arthursu@marshall.edu (S.A.)

**Keywords:** Na/K-ATPase, intestinal absorption, Na-dependent nutrient co-transport, crypt cells, villus cells, cell maturation

## Abstract

Na-K-ATPase on the basolateral membrane provides the favorable transcellular Na gradient for the proper functioning of Na-dependent nutrient co-transporters on the brush border membrane (BBM) of enterocytes. As cells mature from crypts to villus, Na-K-ATPase activity doubles, to accommodate for the increased BBM Na-dependent nutrient absorption. However, the mechanism of increased Na-K-ATPase activity during the maturation of enterocytes is not known. Therefore, this study aimed to determine the mechanisms involved in the functional transition of Na-K-ATPase during the maturation of crypts to villus cells. Na-K-ATPase activity gradually increased as IEC-18 cells matured in vitro from day 0 (crypts) through day 4 (villus) of post-confluence. mRNA abundance and Western blot studies showed no change in the levels of Na-K-ATPase subunits α1 and β1 from 0 to 4 days post-confluent cells. However, Na-K-ATPase α1 phosphorylation levels on serine and tyrosine, but not threonine, residues gradually increased. These data indicate that as enterocytes mature from crypt-like to villus-like in culture, the functional activity of Na-K-ATPase increases secondary to altered affinity of the α1 subunit to extracellular K^+^, in order to accommodate the functional preference of the intestinal cell type. This altered affinity is likely due to increased phosphorylation of the α1 subunit, specifically at serine and tyrosine residues.

## 1. Introduction

An essential function of the mammalian small intestine is nutrient absorption [1]. While the intestinal epithelium is composed of multiple specialized cell types including goblet cells, enteroendocrine cells, Paneth cells, and enterocytes [2], only enterocytes are responsible for nutrient absorption from the intestinal lumen. The enterocytes comprise of undifferentiated crypt cells, which proliferate and differentiate to mature villus cells [3]. Nutrient, electrolyte and fluid absorption primarily occur through the villus cells while the crypt cells are thought to be primarily secretory.

During the differentiation process, enterocytes acquire more transport properties, and are physiologically able to absorb more nutrients compared to undifferentiated crypt cells. Among the acquired transporters, Na-K-ATPase, a basolateral membrane (BLM) transporter, plays a vital role in regulating ionic homeostasis, cell volume and maintaining membrane potential [4]. Na-K-ATPase transports three Na^+^ out of the cell in exchange for two K^+^ into the cell, thus maintaining a high level of intracellular K^+^ and low intracellular Na^+^ concentration [5]. This establishes a Na^+^ gradient that is responsible for driving other secondary transport processes across the brush border membrane (BBM). Transport of different ions (H^+^, Ca^2+^, Cl^–^, PO_4_^2−^, SO_4_^2−^), nutrients like glucose, amino acids and vitamins, certain nucleic acids, bile acids, and neurotransmitters across the plasma membrane are all dependent on Na-K-ATPase activity [6,7]. Previous studies have shown that Na-nutrient co-transport processes in the BBM, namely Na-glucose (SGLT1), Na-alanine (ATB0), Na-glutamine (B0AT1), Na-taurocholate (ASBT) and Na-adenosine (DMT1) are dependent on the BLM Na-K-ATPase for their optimal activity. Furthermore, their inhibition in villus cells in a rabbit model of inflammatory bowel disease (IBD) [8,9,10] is at least in part secondary to altered Na extruding capacity of the cell due to diminished Na-K-ATPase activity. Therefore, Na-K-ATPase plays a crucial role in nutrient absorption and maintenance of intestinal health.

The Na-K-ATPase enzyme consists of various subunits: alpha (α), beta (β) and gamma (γ) subunits [5]. The α and β subunits are ubiquitously present and are important for the activity of Na-K-ATPase [11,12], while the γ-subunit is an optional subunit and its expression is restricted to certain tissues [13]. Of these subunits, α subunit does the catalytic function of the transporter [12], whereas other subunits (β and γ) are responsible for the regulatory function of the α subunit [14,15]. The α subunit is a transmembrane protein having intracellular Na^+^, ATP and phosphate binding sites, and extracellular K^+^ binding sites [5]. The β subunit is important for the proper folding of α subunit and its translocation to the plasma membrane [16,17]. At least four isoforms of α subunit (α1, α2, α3, and α4) and three isoforms of β (β1, β2, and β3) subunit are known. These isoforms are expressed in a tissue-specific manner [5,18,19]. The combination of different isoforms of α and β subunits make up a series of Na-K-ATPase isoenzymes [20]. Each isoenzyme has a different functional property, and they are expressed differentially in tissue- and cell specific-manner [21]. Of these isoforms, α1 and β1 are ubiquitously present in epithelial cells and are also present in the mucosa of the intestine [22].

Na-K-ATPase activity may change according to the physiological requirements of the cell. Several mechanisms may regulate Na-K-ATPase activity. For example, availability of the substrate (Na^+^, K^+^ and ATP), the amount of the enzyme at the plasma membrane, which can be modified by changes in the rate of synthesis or degradation of the individual Na pump polypeptide, and also by the movement of the pump from cytoplasmic vesicles to the plasma membrane by exo/endocytotic vesicular transport [23]. Besides these mechanisms, Na-K-ATPase activity at the cell surface is directly regulated by direct phosphorylation and dephosphorylation by protein kinases and protein phosphatases [24,25,26]. Alterations in the phosphorylation levels of α and regulatory γ subunits are known to change the affinity and, therefore, the activity of the transporter.

Studies using in vivo models have shown that the Na-K-ATPase functional activity varies depending on the cell type and function: rabbit villus cells that have the primary function of sodium-dependent nutrient absorption have twice the amount of Na-K-ATPase function compared to the crypt cells that have minimal absorptive capacity [27]. However, the mechanisms underlying the alteration of Na-K-ATPase function during maturation of crypts to villus cells is not known. Therefore, this study was aimed to determine the mechanism(s) underlying the alteration of Na-K-ATPase activity during growth and maturation of intestinal epithelial cells.

## 2. Results

### 2.1. Alkaline Phosphatase (ALP) Levels during Cell Maturation

**Figure 1:**
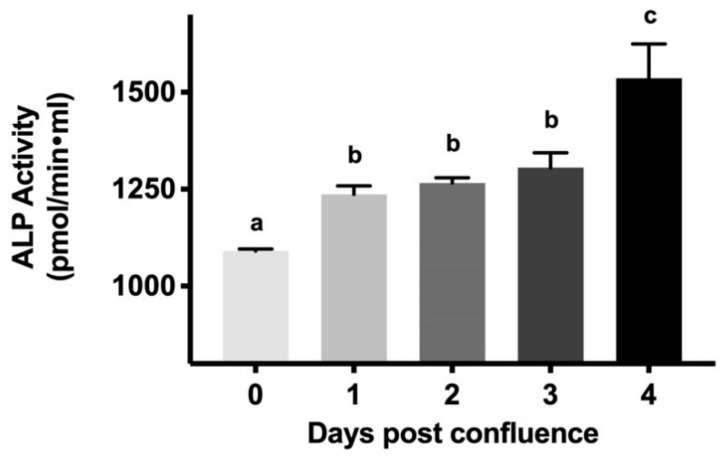
Alkaline phosphatase (ALP) activity to validate IEC-18 cell maturation. Values are represented as means ± standard error of the mean (SEM), *n* = 4. Values not sharing common superscripted letters are significantly different at *p* < 0.001.

To determine cell growth and maturation of intestinal epithelial cells (IEC-18), alkaline phosphatase activity was measured from cells collected from 0-day to 4-day post confluence (Figure 1). The highest level of alkaline phosphatase activity was observed at 4-days post-confluence (1536 ± 44.16 picomole/min·mL), whereas the lowest level of activity (1092 ± 5.432 picomole/min·mL) was observed at 0-day post-confluence. Thus, increasing alkaline phosphatase activity demonstrates that cells mature as they grow from 0-day post confluence (crypt-like) to 4-day post confluence (villus-like).

### 2.2. Na-Dependent Glucose Uptake during Cell Maturation

**Figure 2:**
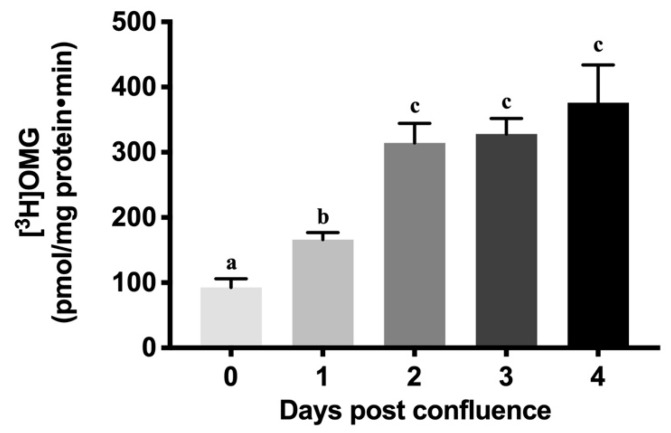
Increase in Na-dependent glucose uptake as IEC-18 cells matured. Uptake was performed in the presence and absence of phlorizin (1 mM) in reaction medium containing [^3^H]-OMG tracer. Values are represented as means ± SEM, *n* = 6 independent experiments. Values not sharing common superscripted letters are significantly different at *p* < 0.001.

As enterocytes mature from crypt to villus, physiological alterations are accompanied by the appearance of different transporters in the BBM. Specifically, the Na-glucose co-transporter SGLT1 appears as enterocytes mature from crypt to villus. Similar to in vivo observations [28], we also found that Na-dependent glucose uptake increased almost three-fold as cells matured from crypt-like to villus-like. Figure 2 shows that minimal SGLT1 activity (92.53 ± 13.35 picomole/mg protein·min) was seen at 0-day post-confluence. There was a steady and robust increase in SGLT1 activity from 0- to 4-day post-confluence in IEC-18 cells (376 ± 57.71). This phenomenon of increasing SGLT1 activity may be due to increasing cellular maturation, cellular polarity and/or an increase in the number of transporters itself, and is comparable to what is seen in vivo during crypt to villus maturation [29].

### 2.3. Na-K-ATPase Activity Levels during Cell Maturation

**Figure 3:**
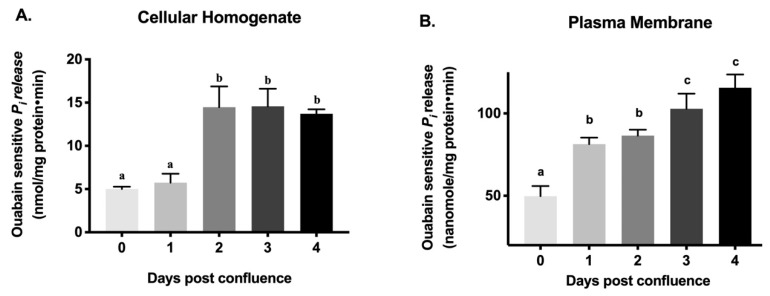
Na-K-ATPase activity as measured by *P_i_* release in IEC-18 cells. Na-K-ATPase activity was measured in the presence or absence of ouabain (1 mM). The absolute Na-K-ATPase activity presented was calculated by subtracting *P_i_* release in the presence of ouabain from that in the absence of ouabain. (**A**) Cellular homogenates. (**B**) Plasma membrane preparations. Values are represented as means ± SEM, *n* = 5. Values not sharing common superscripted letters are significantly different at *p* < 0.01.

**Figure 4:**
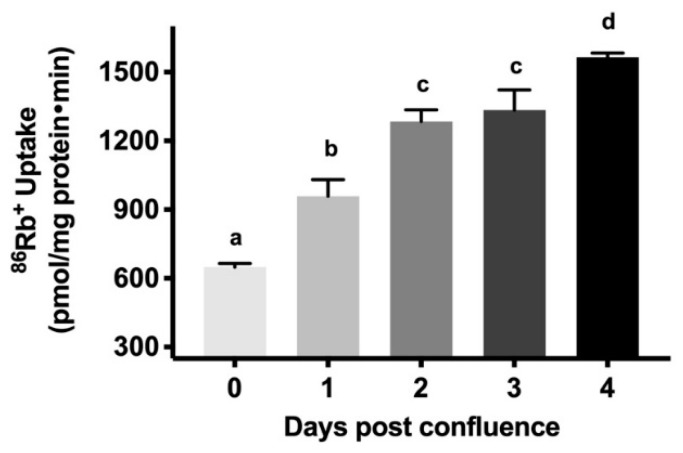
Na-K-ATPase activity as measured by ^86^Rb^+^ uptake in IEC-18 cells. Values are represented as means ± SEM, *n* = 5. Values not sharing common superscripted letters are significantly different at *p* < 0.01.

As enterocytes mature, along with the appearance of BBM transporters, BLM Na-K-ATPase activity increases to provide the favorable Na gradient necessary to absorb nutrients. Similar to the in vivo observation in rabbit intestine [27], Na-K-ATPase activity, as determined by inorganic phosphate (*P_i_*) release, increased gradually in IEC-18 cellular homogenates from 0–4 days post-confluence (Figure 3A: 5.01 ± 0.12 nanomole/mg protein·min in 0-day, 5.74 ± 0.47 in 1-day, 14.48 ± 1.07 in 2-day, 14.58 ± 0.90 in 3-day, 13.72 ± 0.23 in 4-day). Since Na-K-ATPase is functional only as a membrane protein, plasma membrane was prepared from IEC-18 cells and Na-K-ATPase activity was measured. In plasma membrane preparations also, Na-K-ATPase activity increased gradually from 0-day to 4-day (Figure 3B: 49.81 ± 02.74 nanomole/mg protein·min in 0-day, 81.36 ± 1.775 in 1-day, 86.53 ± 1.617 in 2-day, 102.8 ± 4.01 in 3-day, 115.6 ± 3.58 in 4-day). Finally, since radiolabeled rubidium (^86^Rb^+^) uptake is more a functional measure of Na-K-ATPase in live cells, it was also carried out. ^86^Rb^+^ uptake also increased gradually from 0-day to 4-day (Figure 4: 649.2 ± 6.95 nanomole/mg protein·min in 0-day, 958.6 ± 32.52 in 1-day, 1285 ± 22.28 in 2-day, 1335 ± 39.27 in 3-day, 1565 ± 8,17 in 4-day). Thus, these data indicate that as IEC-18 cells mature from crypt-like at 0-day post-confluence to villus-like at 4-day post-confluence, Na-K-ATPase activity increased comparable to in vivo crypt and villus cells.

### 2.4. Kinetic Studies of Na-K-ATPase Activity during Cell Maturation

To determine the mechanism of the increase in Na-K-ATPase activity from 0–4 days post-confluence, we performed ^86^Rb^+^ kinetics in IEC-18 cells. As the concentration of extracellular Rb^+^ was increased, ^86^Rb^+^ uptake was stimulated and subsequently became saturated in all conditions. Kinetic parameters showed that there was no significant change in *V_max_* among the groups. However, there was a significant difference in *K_m_* from 0–4 days post-confluence. Affinity (1/*K_m_*) of Na-K-ATPase activity increased gradually as the cells matured from 0-4 days post-confluence as shown in Table 1.

### 2.5. Na-K-ATPase α1 and Na-K-ATPase β1 Subunit mRNA Abundance during Cell Maturation

**Figure 5:**
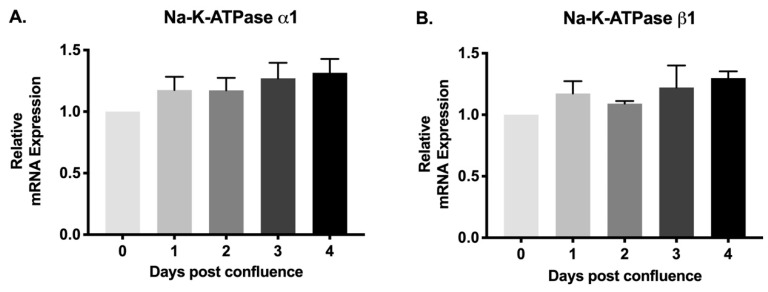
Quantitative real-time polymerase chain reaction (qRT-PCR) analysis of IEC-18 cells on different days of post confluence. Values are relative to 0-day and normalized to β-actin. (**A**). Na-K-ATPase α1. (**B**). Na-K-ATPase β1. Values are represented as mean ± SEM, *n* = 4.

The α subunit primarily provides Na-K-ATPase functional activity whereas the β subunit does not have pumping activity, but contributes for proper transportation of α subunit to the plasma membrane to make the entire protein fully functional. Therefore, to determine whether the change in Na-K-ATPase activity may be transcriptionally regulated, we performed quantitative real-time polymerase chain reaction (qRT-PCR) analysis. There was no significant difference in the relative expression of Na-K-ATPase α1 mRNA (Figure 5A) between different groups (0–4 days). Similarly, the Na-K-ATPase β1 subunit mRNA abundance (Figure 5B) was also not statistically different between the groups (0–4 days).

### 2.6. Na-K-ATPase α1 and Na-K-ATPase β1 Subunit Protein Expression during Cell Maturation

**Figure 6:**
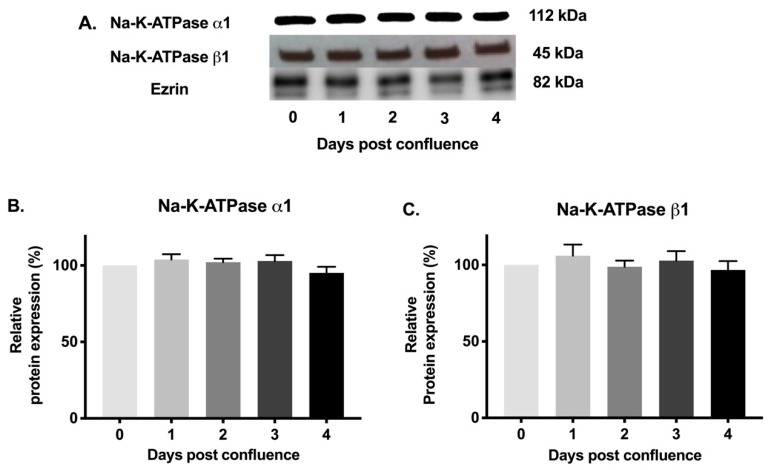
Western blot analysis of IEC-18 cells in cellular homogenates. (**A**). Representative blot of Na-K-ATPase α1, Na-K-ATPase β1 and internal control β-actin. Densitometric quantitation of Western blots. (**B**). Na-K-ATPase α1 and (**C**). Na-K-ATPase β1. Values are relative to 0-day and normalized to β-actin. Values are represented as mean ± SEM, *n* = 4.

**Figure 7:**
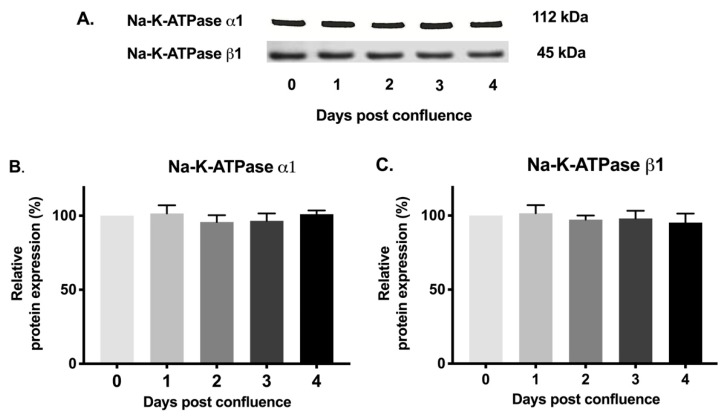
Western blot analysis of IEC-18 cells in plasma membrane fractions. (**A**). Representative blots of Na-K-ATPase α1 and Na-K-ATPase β1. Densitometric quantitation of Western blots. (**B**). Na-K-ATPase α1 and (**C**). Na-K-ATPase β1. Equal amount of proteins were loaded. Values are relative to 0-day and represented as mean ± SEM, *n* = 4.

Since mRNA levels of Na-K-ATPase α1 and Na-K-ATPase β1 subunits may not definitely correlate with protein expression, Western blot analysis of protein expression of Na-K-ATPase α1 and Na-K-ATPase β1 subunits were performed. Immunoreactive protein levels of Na-K-ATPase α1 and Na-K-ATPase β1 subunits were determined in cellular homogenates and plasma membrane fractions. Densitometric analysis of relative protein expression revealed that the level of Na-K-ATPase α1 protein expression was not statistically different in IEC-18 cells from 0–4 days post-confluence in cellular homogenates (Figure 6A,B) or plasma fractions (Figure 7A,B). Similarly, there was also no significant difference in Na-K-ATPase β1 subunit protein expression among IEC-18 cells from 0–4 days post-confluence in cellular homogenates (Figure 6A,C) and plasma membrane fractions (Figure 7A,C). Protein expression of Na-K-ATPase α1 and Na-K-ATPase β1 subunits correlates with mRNA expression of their respective subunits. Furthermore, these studies are consistent with kinetic parameters determined above. Therefore, the expression of Na-K-ATPase α1 and Na-K-ATPase β1 subunits are unaltered in cellular homogenates and plasma membrane fractions in IEC-18 cells during maturation. Thus, the increase in Na-K-ATPase activity as IEC-18 cells mature from crypt-like to villus-like cells is secondary to increased affinity of the protein, which may be due to altered phosphorylation of the α1 Na-K-ATPase subunit.

### 2.7. Protein Phosphorylation Levels of Na-K-ATPase α1 Subunit during Cell Maturation

**Figure 8:**
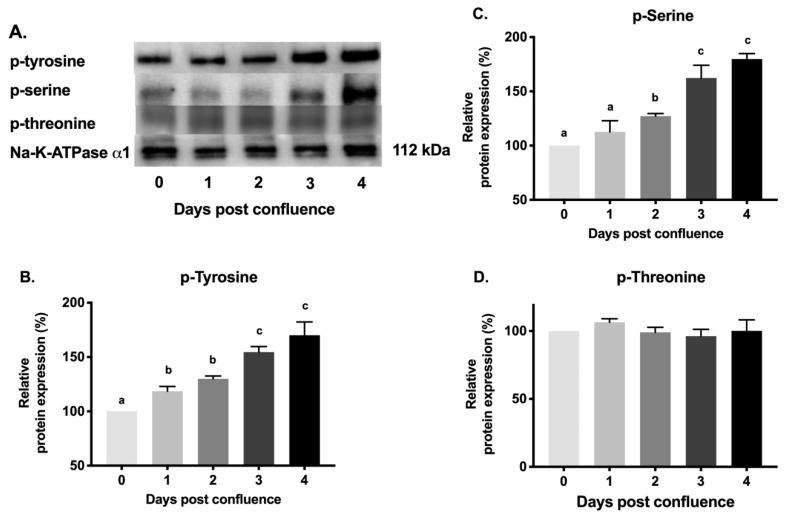
Western blot analysis of phosphorylation levels of Na-K-ATPase α1 in plasma membrane during different days post confluence. (**A**). Representative blot of p-tyrosine, p-serine, p-threonine and Na-K-ATPase α1 after immunoprecipitation with Na-K-ATPase α1 antibody. Densitometric quantitation of Western blots. (**B**). p-tyrosine. (**C**). p-Serine. (**D**). p-Threonine. Values are relative to 0-day and normalized to Na-K-ATPase α1. Values are represented as mean ± SEM, *n* = 3. Values not sharing common superscripted letters are significantly different at *p* < 0.05.

**Figure 9:**
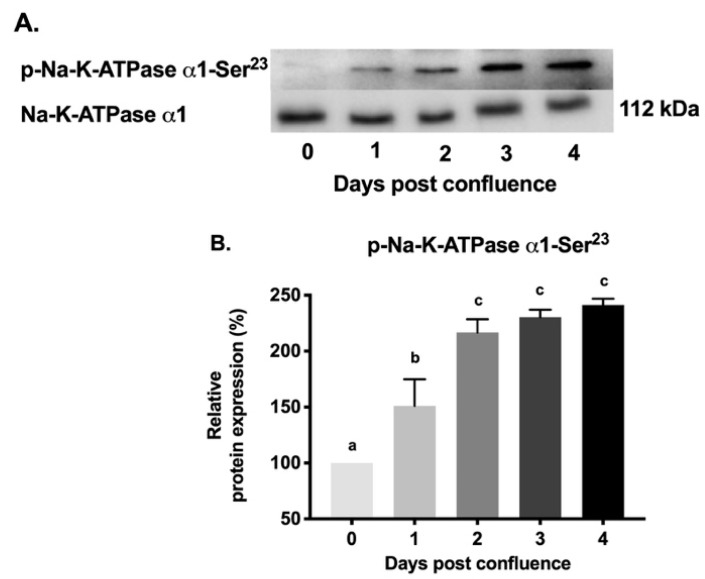
Western blot analysis of plasma membrane Na-K-ATPase α1-Ser23 at different days of post confluence. (**A**). Representative blot of experiment done in triplicate. (**B**). Densitometric analysis of Western blots. Values are relative to 0-day and normalized to Na-K-ATPase α1. Values are represented as mean ± SEM, *n* = 3. Values not sharing common superscripted letters are significantly different at *p* < 0.01.

To examine whether changes in Na-K-ATPase activity during different days of post confluence may be due to alteration in phosphorylation of Na-K-ATPase α1 subunit, thus affecting its affinity, immunoprecipitation studies were performed. Na-K-ATPase α1 subunit was immunoprecipitated from the plasma membrane on different days of post-confluence and probed with antibodies specific for phosphorylated amino acids (serine, threonine, and tyrosine). As shown in the representative blot and densitometric analysis in Figure 8, immunoprecipitated Na-K-ATPase α1 subunit from different days of post confluence had a gradual increase in the levels of phosphorylated tyrosine (Figure 8A,B; 100% in 0-day and 170 ± 21.41 in 4-day post-confluence). Similarly, phosphorylated serine levels also increased (Figure 8A,C; 100% in 0-day and 179 ± 5.08 in 4-day post confluence). However, phosphorylated threonine levels remained unchanged (Figure 8A,D). To determine which specific residues might be the target of phosphorylation during maturation, protein extracted from different days were probed with anti-p-Na-K-ATPase α1-Ser^23^ antibody, the target of which has been previously implicated in alterations in Na-K-ATPase activity. As shown in the representative blot and densitometric analysis in Figure 9, p-Na-K-ATPase α1-Ser^23^ had a gradual increase (100% in 0-day and 241.3 ± 5.5 in 4-day post-confluence) in its phosphorylation levels during IEC-18 cell maturation. These data confirmed that change in Na-K-ATPase activity during cell maturation is likely due to changes in phosphorylation of Na-K-ATPase α1 subunit, specifically at serine and tyrosine amino acid residues and possibly involving p-Na-K-ATPase α1-Ser^23^ residue.

## 3. Discussion

Villus and crypt cells have been extensively investigated concerning absorption mechanisms of Na-dependent nutrient co-transporters at the level of the co-transporters in the BBM. At the cellular level, all of these Na-dependent co-transport processes are regulated secondary to alterations in BLM Na-K-ATPase since it provides the necessary Na gradient for their secondary active transport processes. During the maturation of enterocytes from crypts to villus, Na-K-ATPase activity doubles to accommodate for the increased BBM Na-dependent nutrient absorption by the villus cells. However, the Na-K-ATPase located in the BLM that drives these nutrient transporters have not been previously studied for their capacity to adapt themselves to support the increasing nutritional absorptive capacity that the intestinal epithelial cells acquire during their growth and maturation. Therefore, the present study was undertaken to understand the mechanism of Na-K-ATPase regulation during the maturation of intestinal epithelial cells from the crypt to the villus.

During the maturation of intestinal cells from the crypt to the villus, significant morphological and physiological changes occur. Morphological changes include changes in composition and increases in brush border membrane surface area. Along with these changes, physiologically mature cells acquire more transporters and, therefore, their capacity to absorb nutrients increases. A study of the crypt-villus axis of piglets has shown that there is upregulation of proteins associated with glycolysis/gluconeogenesis, fatty acid metabolism, amino acid metabolism, and citrate cycle as cells mature from crypt to villus [30]. Additionally, it has been reported that alkaline phosphatase (ALP), aminopeptidase, sucrose, lactase, and Na-K-ATPase activities increased along the crypt-villus axis in jejunum and ileum of neonatal pigs [31]. Similar to these results, our study has shown that there is an increase in ALP and SGLT1 activities as IEC-18 cells mature from crypt-like to villus-like cells in vitro [30,31].

There are conflicting reports in the literature addressing the levels of Na-K-ATPase activity during the maturation of cells [32,33,34]. For example, in one study a reduction in activity was found to be due to the shedding of Na-K-ATPase into exosomes or due to degradation [35]. The present study, however, has shown that there is an increase in Na-K-ATPase activity as IEC-18 cells mature from crypt-like to villus-like cells. The increase or decrease in activity may be the result of various mechanisms: firstly, due to an increase or decrease in the protein levels of the subunits α and β [36,37,38,39]. The increased Na-K-ATPase activity could, therefore, be due to increase in the protein levels of these subunits by increased mRNA and protein synthesis. Secondly, increased translocation of alpha and beta subunits from the cytoplasm to the plasma membrane can increase Na-K-ATPase activity [24,25,40]. Among different isoforms of α and β subunits, α1 and β1 isoforms are expressed ubiquitously and are known to perform a housekeeping role in most epithelial cell types including intestinal epithelial cells [22,41]. During the development of neonates to newborn, Na-K-ATPase enzyme activity in erythrocytes was found to decrease with maturation, correlating with a decrease in α1 subunit [42]. Similarly, in conjunction with Na-K-ATPase activity, the α1 isoform also increased during the maturation of cochlea [43]. However, in the present study, we did not find any significant changes in mRNA expression of the α1 isoform or its protein levels although there was an increase in Na-K-ATPase activity.

For Na-K-ATPase to be fully functional as a transporting unit in the plasma membrane, assembly of β subunit with α subunit is essential. The association of β subunit with α subunit is required for maturation, membrane insertion and transport activity of the alpha subunit [44]. Na-K-ATPase α and β heterodimer are selective in their subunit isoform association, where α1 mostly assembles with β1 isoform [44]. Although there was elevated Na-K-ATPase activity, the expression levels of α1 and β1 mRNA and protein in cellular homogenate remain unchanged during maturation. Therefore, the level of Na-K-ATPase α1 and β1 mRNA and protein in cellular homogenate confirmed that Na-K-ATPase is regulated post-translationally. Correspondingly, there was also no alteration in Na-K-ATPase α1 and β1 expression in the plasma membrane. Therefore, the unaltered level of Na-K-ATPase α1 and β1 protein expression in both cellular homogenate and plasma membrane also indicate that increased Na-K-ATPase activity during maturation is not likely due to altered membrane trafficking.

The increase in Na-K-ATPase activity could be due to change in the affinity of Na-K-ATPase for Na^+^, K^+^ and ATP [45,46]. In the present study, there was an increase in the affinity for K^+^ of Na-K-ATPase during cell maturation. This change in affinity may be due to alteration in the level of the phosphorylation of the protein. Considerable evidence indicates that the Na-K-ATPase activity is regulated by phosphorylation of Na-K-ATPase α1 subunit on serine, threonine [47,48,49,50] and tyrosine residues [51,52]. It has been reported that there are at least 21 phosphorylation sites in rat Na-K-ATPase α1 [53], including 6 tyrosine, 1 threonine, and 14 serine phosphorylation sites. Protein kinases A and C (PKA, PKC) have been reported to phosphorylate α1 subunit specifically on serine and threonine residues [47,48,49,50], whereas various tyrosine kinases phosphorylate at tyrosine residues thus regulating Na-K-ATPase activity [51,52]. Multiple studies have reported that the phosphorylation of the Na-K-ATPase α1 subunit on tyrosine residues causes different responses on Na-K-ATPase activity [51,52,54]. While some reports have demonstrated that increased tyrosine phosphorylation of Na-K-ATPase α1 in rat kidney tubules [51] and human skeletal muscle cells [54] increase the activity of the Na-K-ATPase, others have reported that increased tyrosine phosphorylation of Na-K-ATPase α1 in human and pig renal cells reduces the activity of Na-K-ATPase [52]. This differential regulation of Na-K-ATPase pump activity by tyrosine phosphorylation might be due to phosphorylation at different amino acid residues or due to the differences in both tissue and species. Our study demonstrates that there is an increase in Na-K-ATPase α1 tyrosine phosphorylation in conjunction with its increase in activity as cells mature. The different results suggest that, depending on the tissue studied, and the experimental conditions, phosphorylation of serine and tyrosine leads to either activation or inhibition of Na-K-ATPase [40]. However, in the present study, we observed that there was an increase in serine and tyrosine phosphorylation whereas there was no change in threonine phosphorylation of Na-K-ATPase α1, as cells matured.

## 4. Materials and Methods

### 4.1. Cell Culture

Rat small intestinal epithelial cells (ATCC CRL-1589, American Type Culture Collection, Manassas, VA, USA), between passages 5 and 20, were used for all of the experiments. Cells were maintained in Dulbecco’s modified Eagle’s medium (DMEM), supplemented with 10% (*v*/*v*) fetal bovine serum, 100 U/L human insulin, 0.25 mM β-hydroxybutyric acid and 100 U/mL of penicillin and streptomycin. These cells were cultured in a humidified atmosphere of 10% CO_2_ at 37 °C. The cells were fed with fresh medium every other day. When the cells reached 100% confluence, it was considered as 0 day, and cells were grown until 4 days post-confluence. Experiments were conducted on 0-day, 1-day, 2-day, 3-day, and 4-day post-confluent cells.

### 4.2. Alkaline Phosphatase Activity (ALP) Assay

The activities of ALP were determined using an enzyme assay kit, according to the manufacturer’s protocol (Alkaline phosphatase assay kit, colorimetric (Abcam#83369) Abcam PLC, Cambridge, MA, USA). ALP activity was calculated as picomole/min/mL from the cells that were harvested between 0 and 4-day post-confluence.

### 4.3. Na-Dependent Glucose Uptake Studies

To determine Na-dependent glucose transport in IEC-18 cells, glucose uptake studies were performed on cells grown on transwell inserts (0.4 μm; #140620, Thermo Fisher Scientific Inc., Waltham, MA, USA) in 24 well plates. Cells were rinsed once with warm wash buffer (130 mM NaCl, 5 mM KCl, 1mM MgSO_4_, 2 mM CaCl_2_, 20 mM HEPES [4-(2-Hydroxyethyl)-1-poperazineethanesulfonic acid] and pH adjusted to 7.4) and incubated with the same buffer for 10 min. After the incubation, wash buffer was removed from the wells and replaced with the reaction mixture containing a trace amount of [^3^H] OMG in Na-HEPES buffer with and without phlorizin (SGLT1 inhibitor). The reaction was stopped after two minutes with cold wash buffer (130 mM NaCl, 5mM KCl, 1mM MgSO_4_, 2 mM CaCl_2_, 20 mM HEPES, 10 mM D-Glucose and pH adjusted to 7.4), and the cells were then washed twice with the same buffer. Cells were lysed by incubating them with 1M NaOH for 20 min at 70 °C. The lysed cells from each well was then mixed with 5 mL of Ecoscint A (National Diagnostics, Atlanta, GA, USA). The vials were kept in darkness overnight and radioactivity retained by the cells was determined in a Beckman Coulter 6500 scintillation counter (Beckman Coulter Inc., Brea, CA, USA).

### 4.4. Crude Plasma Membrane Isolation

Plasma membrane (crude) was prepared from cells according to the method of Havrankova et al. [55]. Briefly, 100 mg cells were homogenized in 5 mL of 1 mM NaHCO_3_ and centrifuged at 600× *g* for 30 min. The supernatant was collected and centrifuged for 30 min at 20,000× *g*. After washing membrane pellets with 1mM NaHCO_3_ for a couple of times_,_ final pellet was resuspended in 0.04 M Tri-HCl buffer (pH 7.4) containing 0.1% bovine serum albumin (BSA). All the procedures mentioned above were carried out at 4 °C.

### 4.5. Na-K-ATPase Activity Assay

Na-K-ATPase activity was measured as *P_i_* liberated in cellular homogenates and crude plasma membrane fractions from the cells that were harvested 0-4 days post-confluence according to the protocol of Forbush et al. [56]. Enzyme-specific activity was expressed as nanomoles of *P_i_* released per milligram protein per minute.

### 4.6. Determination of Na-K-ATPase Activity and Kinetics by ^86^Rb^+^ Uptake

Uptake studies were performed in cells grown on transwell inserts in 24 well plates. IEC-18 cells were plated with approximately 1 × 10^5^ cells. Uptake studies for Na-K-ATPase were done using radioactive Rubidium (^86^Rb^+^; PerkinElmer, Inc., Waltham, Massachusetts, U.S.). Cells were incubated for 1 hr in serum-free DMEM media at conditions as mentioned above. The cells were subsequently washed with serum-free media and incubated for 10 min at 37 °C with 20 μM monensin (in serum-free DMEM) added to both the apical and basolateral sides. The cells were then washed with serum-free DMEM. Uptake of ^86^Rb^+^ was started by incubating the cells for 15 min in a reaction mixture (serum-free DMEM) containing ~1 μCi/ of ^86^Rb^+^ added to the basolateral side of the cells in the presence and absence of ouabain (1mM).

For Na-K-ATPase kinetics, after incubation with serum-free DMEM for 1hr, the cells were subsequently washed with incubation buffer (130 mM NaCl, 5 mM KCl, 1 mM MgSO_4_, 2 mM CaCl_2_ and 20 mM HEPES) and incubated for 10 min at 37 °C in the incubation buffer with 20 μM monensin on both sides of the membrane. Then cells were washed with wash buffer (130 mM NaCl, 1 mM MgSO_4_, 2 mM CaCl_2_, 20 mM HEPES and 5 mM mannitol) on both sides. Na-K-ATPase kinetic studies were then performed by incubating the cells for 30 s with reaction mixtures (130 mM tetramethylammonium chloride, 1 mM MgSO_4_, 2 mM CaCl_2_, 20 mM HEPES, 5 mM mannitol and ^86^Rb^+^ (~1 μCi/ well) containing different concentrations (0.05–2 mM) of RbCl added to the basolateral side of the membrane, in the presence and absence of ouabain (1 mM).

For both uptake and kinetics experiment, the reaction was then stopped by the addition of ice-cold MgCl_2_. The cells were then washed three times with MgCl_2_ and lysed with 800 μL of 1N NaOH by incubating them for 30 min at 70 °C. The lysed cells from each well were then mixed with 5 mL of Ecoscint A (National Diagnostics, Atlanta, GA, USA). The vials were kept in darkness overnight and radioactivity retained by the cells was determined in a Beckman Coulter 6500 scintillation counter. The net Na-K-ATPase activity was calculated by subtracting ^86^Rb^+^ uptake in the presence of ouabain from that in the absence of ouabain.

### 4.7. RNA Isolation and Quantitative Real-Time Polymerase Chain Reaction (qRT-PCR)

RNA was isolated from 0–4 days post-confluent cells by using the RNeasy mini kit obtained from Qiagen Inc. (Germantown, MD, USA). qRT-PCR was performed using isolated total RNA by a two-step method. First, total RNA was used to synthesize cDNA using SuperScript III (Invitrogen Life Technologies, Carlsbad, CA, USA). Then, newly synthesized cDNA was used as a template to perform real-time PCR using TaqMan universal PCR master mix from Applied Biosystems (Foster City, CA, USA) according to the manufacturer’s protocol. Rat-specific Na-K-ATPase α1 and Na-K-ATPase β1 primers and probes were used for the qRT-PCR studies. Also, the rat-specific β-actin primer was used to normalize the expression levels of mRNA in the samples.

### 4.8. Immunoprecipitation

Immunoprecipitation (IP) assays were conducted to investigate the phosphorylation level of Na-K-ATPase α1 present in the plasma membrane. Cells were first rinsed with PBS, scraped and collected in PBS, and pelleted by brief low-speed centrifugation (800 rpm). Then collected pellet was further used for plasma membrane isolation as mentioned above. The prepared plasma membrane fraction was sonicated and dissolved in 200 uL of lysis buffer (T-per Buffer; Thermo Fisher Scientific Inc., Waltham, MA, USA) with protease inhibitors (Thermo Fisher Scientific Inc., Waltham, MA, USA). Plasma membrane protein was further diluted to 1 μg/μL in a microcentrifuge tube with T-per buffer and placed on a rocking shaker at 4 °C with 2 μg of Anti-Na-K-ATPase α1 antibody overnight. Antibody-protein complexes formed were captured by adding 50 μL of washed protein G agarose bead slurry (Catalog # 16-266; Thermo Fisher Scientific Inc., Waltham, MA, USA) with rotation at 4 °C for 2 h. The agarose beads with the protein–antibody complex were collected by centrifugation (14,000× *g*) and washed three times with ice-cold lysis buffer. The agarose beads were resuspended in 2× sample loading buffer and incubated at 37 °C for 15 min. The beads in sample loading buffer were vortexed, and the supernatant was used for Western blot analysis.

### 4.9. Western Blot Analysis

Western blot analysis was performed in whole cell lysate and plasma membrane preparations. Equal amounts of protein (20 μg) were denatured in sample buffer (Laemmli sample buffer, BIO-RAD #3100010639; Bio-Rad Laboratories, Hercules, CA, USA) and separated by electrophoresis on an 8%–12% gradient gel. Proteins on the gel were transferred to a polyvinylidene fluoride membrane which was blocked with 5% dry milk or BSA in TBS (20 mM Tris pH 7.5, 150 mM NaCl) with 0.1% Tween-20 and then incubated with one of the following primary antibodies overnight at 4 °C: Na-K-ATPase α1 (Millipore, # 05-369); Na-K-ATPase β1 (ab2873; Abcam PLC, Cambridge, MA, USA); p-Serine (ab9332; Abcam PLC, Cambridge, MA, USA); p-Threonine (ab179530; Abcam PLC, Cambridge, MA, US); p-Tyrosine (ab9337; Abcam PLC, Cambridge, MA, US); p-Na-K-ATPase α1-Ser^23^ (Cell Signaling Technology, #4006) and p-Na-K-ATPase α1-Ser^16^ (Cell Signaling Technology, #4020). The membrane was washed three times with Tris Buffered Saline with Tween 20 (TBST) followed by TBS. The membrane was then incubated with secondary antibody for 1hr. ECL western blotting detection reagent (GE Healthcare Bio-Sciences, Piscataway, NJ, USA) was used to detect the immobilized protein. The chemiluminescence was detected using a FluorChem M instrument (Alpha Innotech, San Leandro, CA, USA) and its software analyzed the intensity of the bands. β-actin was used to normalize the expression levels in cellular homogenate.

### 4.10. Protein Determination

Total protein was measured by the Lowry method using the Bio-Rad protein assay kit (Hercules, CA, USA). BSA was used as a standard.

### 4.11. Statistical Analysis

All groups presented have at least *n* = 3 per group repeated with a different passage. The values are presented as mean ± standard error of the mean (SEM), and significant values of *p* < 0.05 or less were taken to indicate statistical significance. All of the data were analyzed using one-way or two-way analysis of variance (ANOVA) using GraphPad Prism 7 (GraphPad Software Inc., San Diego, CA, USA).

## 5. Conclusions

In summary, this study demonstrated that as epithelial cells mature from secretory crypt-like to absorptive villus-like cells, the Na-K-ATPase activity increases to support the increasing absorptive capacity of the enterocytes. Na-K-ATPase activity increased secondary to increases in affinity rather than an increase in transporter numbers as cells matured from crypt to villus. Specifically, altered affinity was found to be likely due to increased phosphorylation of α1 subunit of Na-K-ATPase.

## Figures and Tables

**Table 1:** Kinetic parameters of Na-K-ATPase transporters in IEC-18 cells.

	0 Day	1 Day	2 Day	3 Day	4 Day
*V_ma_*_x_ (picomole/mg protein·30 S)	742.5 ± 62	714 ± 83.5	718 ± 58.4	713 ± 27.9	733 ± 44.8
*K_m_* (mM)	0.68 ± 0.11a	0.61 ± 0.15a	0.39 ± 0.08b	0.37 ± 0.04b	0.33 ± 0.06b

Uptake of ^86^Rb^+^ as a function of varying concentration of extracellular Rb^+^ (RbCl). As extracellular Rb^+^ concentration was increased, uptake of ^86^Rb^+^ was stimulated and subsequently became saturated in all groups. Kinetic parameters [maximal uptake (*V_max_*) and affinity (1/*K_m_*)] were obtained after the analysis of data with GraphPad Prism 7 software (GraphPad Software Inc., San Diego, CA, USA). Values are represented as mean ± SEM, *n* = 6. Values not sharing common superscripted letters are significantly different at *p* < 0.01.
